# Tolvaptan Improves Contrast-Induced Acute Kidney Injury

**DOI:** 10.1155/2022/7435292

**Published:** 2022-01-30

**Authors:** Chunyang Xu, Xu Huang, Gaoliang Yan, Dong Wang, Meijuan Hu, Chengchun Tang

**Affiliations:** ^1^Department of Cardiology, Zhongda Hospital, School of Medicine, Southeast University, Nanjing, Jiangsu 210009, China; ^2^Department of Cardiology, Yancheng First Hospital, Affiliated Hospital of Nanjing University Medical School (Yancheng No. 1 People's Hospital), Yancheng, Jiangsu 224006, China

## Abstract

**Objective:**

Contrast-induced acute kidney injury (CI-AKI) is a serious side effect of contrast media use. The purpose of this study was to investigate the role and mechanism of tolvaptan (TOL) in CI-AKI.

**Methods:**

24 Wistar male rats were randomly divided into 4 groups (*n* = 6). And a rat model of CI-AKI was established. Then, the blood and urine of rats in each group were collected to detect relevant parameters. HE staining was utilized for the observation of the pathological changes of rat kidney tissues, TUNEL assay for the detection of tubular cell apoptosis, biochemical detection for the confirmation of oxidative stress level in kidney tissues, and western blot for the test of the expression of apoptotic proteins and the Nrf2 signaling pathway-related proteins in kidney tissues.

**Results:**

TOL could significantly reduce the serum level of urea nitrogen, creatinine, and neutrophil gelatinase-associated lipocalin and decrease serum Cys-C and urine KIM-1 in CI-AKI rats. The result above meant that TOL could improve kidney injury and reduce tubular cell apoptosis in CI-AKI rats. In addition, TOL contributed to a reduction of oxidative stress level by downregulating myeloperoxidase level and increasing the activities of superoxide dismutase and glutathione peroxidase in the kidney tissue of CI-AKI rats. After the pretreatment of TOL, the expression of proapoptotic proteins cleaved-caspase 3 and BAX, as well as mitochondrial fusion proteins DRP1 and MFN2 was downregulated, while the expression of Bcl-2 and PINK1 was upregulated in the kidney tissue of CI-AKI rats. Further, TOL could activate the Nrf2 signaling pathway, and the Nrf2 inhibitor ML385 reversed the effect of TOL on CI-AKI.

**Conclusion:**

TOL can improve CI-AKI by activating the Nrf2/HO-1 signaling pathway, inhibiting oxidative stress response, and reducing tubular cell apoptosis.

## 1. Introduction

Contrast-induced acute kidney injury (CI-AKI), also known as contrast-induced nephropathy (CIN), refers to an acute renal complication occurring after the application of contrast media. CI-AKI can cause temporary or permanent renal failure, which is generally defined as a decrease in renal function within 24-72 h after the application of contrast media [1]. Percutaneous coronary intervention (PCI) has become one of the important causes of hospital-acquired acute kidney injury (AKI); specifically, extensive application of PCI has caused large number of use of contrast media, thus increasing the number of CI-AKI cases [2]. Usually, the incidence of CI-AKI is low, often below 10%, but the incidence can be significantly increased in high-risk groups, even to more than 50% [3]. CI-AKI remains an important cause of overall mortality, prolonged hospitalization, and increased economic burden [4]. And renal insufficiency caused by CI-AKI can seriously affect the quality of life of patients and can even lead to disability [5]. Therefore, it is essential to study CI-AKI and find effective prevention and treatment strategies. The pathogenesis of CI-AKI is complex and poorly understood but is mainly characterized by renal vasoconstriction, tubular cytotoxicity, and medullary ischemia due to reactive oxygen species (ROS) formation [6, 7]. Studies have shown that diuretics combined with intravenous infusions can increase urine volume thereby diluting the contrast media in the renal tubules and also can protect renal medullary ischemia [8, 9].

Tolvaptan (TOL) is a prototypical antidiuretic hormone V2 receptor antagonist that increases free water excretion [10]. After competitive binding of TOL to V2 receptors, aquaporin-2 is shed from the inner membrane of the collecting ducts, which leads to a reduction in reabsorption of free water and an increase in the excretion of free water in the urine, but the excretions of urinary sodium and potassium were not increased [11]. Therefore, TOL can reduce body water retention and patient weight without significant electrolyte loss, thereby increasing serum sodium concentration and reducing urine osmotic pressure [12]. However, the effect of TOL on renal function remains controversial. Some studies have reported elevated serum creatinine (Cr) in patients treated with TOL [13, 14]. By contrast, Shirakabe et al. [15] demonstrated that early application of TOL in acute heart failure prevented AKI and improved the prognosis of patients. A case reported by Lee et al. [16] also showed that TOL could rescue CI-AKI patients and prevent hemodialysis. And it is also reported that TOL was effective in preventing AKI in patients with contrast-induced heart failure and chronic kidney disease [17]. All of the above studies have confirmed that TOL is effective in preventing and treating patients with CI-AKI. However, no studies have reported the mechanism of the effect of TOL on CI-AKI. Therefore, in this study, a rat model of CI-AKI was constructed to observe the protective effect and investigate specific molecular mechanism of TOL on kidney injury.

## 2. Materials and Methods

### 2.1. Animals

Adult male Wistar rats (Age: 8 weeks; weight: 250-350 g) were purchased from Guangdong Medical Experimental Animal Center. Then, the rats were adaptively housed in an environment with a relative humidity of 60%, a temperature of 22°C, and a cycle of 12 h light/12 h dark. During the feeding, the rats were allowed to drink and eat freely. This study was approved by the Ethics Committee of Guangdong Medical Experimental Animal Center (C202109-17).

### 2.2. Rat Model of CI-AKI

**Figure 1:**
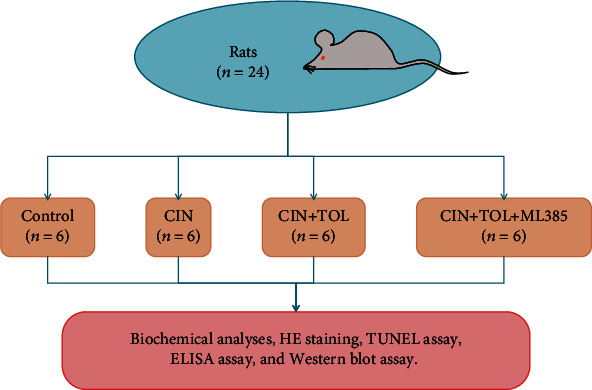
Flow diagram of animal experiments. CIN: contrast-induced nephropathy; TOL: tolvaptan; ML385: Nrf2 inhibitor.

The rat model of CI-AKI was established as the way described previously [18] . Twenty-four rats were randomly divided into four groups (6 rats/group). In the control group, rats were injected with the same amount of saline in the tail vein. In the CIN group, rats were injected with 10 mg/kg indomethacin in the tail vein, followed by 10 mg/kg NG-nitro-L-arginine methyl ester (dissolved in saline) 15 min later. Then, after another 15 min, ioversol was injected (8.3 mL/kg of organically bound iodine). In the CIN + TOL group, intragastric administration of 10 mg/kg TOL [19] was performed 48 h before induction of CI-AKI. In the CIN + TOL + ML385 group, rats were received intragastric administration of 10 mg/kg TOL and intraperitoneal administration of 10 mg/kg Nrf2 inhibitor ML385 48 h before induction of CI-AKI. Twenty-four h after induction of CI-AKI, rats were anesthetized with 2% sodium pentobarbital (30 mg/kg). And then the kidney tissue, blood, and urine were collected from each rat. The modeling process is shown in Figure 1.

### 2.3. Detection of Serum Biochemical Parameters

First, 2 mL of blood was collected and placed in a blood collection tube for centrifugation (3500 rpm, 15 min, 4°C) to separate serum. Then, the serum was stored at -80°C for follow-up detection. The final step was to confirm the level of blood urea nitrogen (BUN; C013-2-1, Nanjing Jiancheng Bioengineering Institute, China), sCr (C011-2-1), and neutrophil gelatinase-associated lipocalin (NGAL; H392-1) according to the instructions of the assay kits.

### 2.4. Detection of Activities of Myeloperoxidase (MPO), Superoxide Dismutase (SOD), and Glutathione Peroxidase (GSH-Px)

On completion of separation of renal capsule, the left kidney of each rat was placed in a cryotube for rapid storage into liquid nitrogen. Then, the samples were transferred to a -80°C freezer for storage. For detection, the frozen tissues were washed with precooled normal saline, dried with filter paper, and then were weighed. After that, the tissues were utilized for the preparation of 10% of tissue homogenate by adding normal saline according to the weight-to-volume ratio. Then, under the ambient temperature, the centrifugation was conducted at 3000 rpm for 10 min. The supernatant was extracted to determine the protein concentration using Coomassie Brilliant Blue (Gibco, USA) and to assess the activities of MPO, SOD and GSH-Px according to the instruction of kits (Nanjing Jiancheng Bioengineering Institute, China).

### 2.5. Hematoxylin-Eosin (H&E) Staining

The right kidney of rats was immersed in 10% of formaldehyde solution for fixation. Then, the samples were dehydrated, immersed in wax, and embedded in paraffin to prepare paraffin sections (5 *μ*m). After that, the sections were stained with hematoxylin and eosin. The stained sections were cleared by xylene and finally mounted with neutral gum. The kidney tissues were placed under a light microscope to observe the pathological changes. Histopathology scoring was detected as previously reported [20]. The scoring system for 10 randomly selected nonoverlapping areas reflecting tubular necrosis, cast formation, loss of brush border, and tubular dilatation was as follows: 0 (none), 1 (≤10%), 2 (11-25%), 3 (26-45%), 4 (46-75%), and 5 (76-100%).

### 2.6. TdT-Mediated dUTP-Biotin Nick End Labeling (TUNEL) Staining

Apoptosis was measured by the TUNEL method [21]. On completion of fixation, embedding, sectioning, and deparaffinization of kidney tissues, 20 *μ*g/mL proteinase *K* was added dropwise. After 30 min, 50 *μ*L TUNEL solution was added, followed by incubation for 60 min at 37°C. Finally, the sections were mounted, observed, and analyzed under a fluorescence microscope.

### 2.7. Enzyme-Linked Immunosorbent Assay (ELISA)

The level of Cys-C in rat serum and KIM-1 in urine was measured, and all procedures were performed in strict accordance with the instructions of the ELISA kits (Multi Sciences).

### 2.8. Western Blot

The kidney tissues were lysed using RIPA lysis buffer (Beyotime Biotechnology, Shanghai, China) to obtain proteins [22]. After the protein concentration was determined with the BCA kit (Beyotime Biotechnology, Shanghai, China), the proteins were separated by 10% of SDS-PAGE, followed by transferring to the polyvinylidene fluoride (PVDF) membranes. Then, the membranes were blocked in blocking solution for 1 h at ambient temperature. The membranes were then incubated overnight at 4°C with primary antibodies provided by Cell Signaling, Boston, USA, including *β*-actin (#4970S, 1 : 1000), cleaved-caspase3 (#9661S, 1 : 1000), BAX (#5023S, 1 : 1000), Bcl-2 (#15071S, 1 : 1000), DRP1 (#8570S, 1 : 1000), MFN2 (#11925S, 1 : 1000), PINK1 (#6946S, 1 : 1000), Nrf2 (#12721S, 1 : 1000), Keap-1 (#8047S, 1 : 1000), and HO-1 (#86806S, 1 : 1000). The next day, after being rinsed for three times (10 min/time), the membranes were incubated with secondary antibodies (1 : 5000, Beijing ComWin Biotech Co., Ltd., Beijing, China) for 1 h at ambient temperature, followed by a rinsing step for another three times (10 min/time). After dropping the developer on the membranes, the detection was performed using a chemiluminescence imaging system (Bio-rad).

### 2.9. Statistical Analysis

Statistical analyses were performed using IBM SPSS Statistics for Windows, version 21.0 (IBM Corp., Armonk, N.Y., USA), and measurement data were expressed as mean ± standard deviation (SD). For comparison of data among groups, one-way analysis of variance (ANOVA) was applied, with Tukey's test for post hoc analysis. Student's *t*-test was adopted for comparison between groups. A statistically significant difference was suggested if *P* < 0.05.

## 3. Results

### 3.1. TOL Attenuates CI-AKI

**Figure 2:**
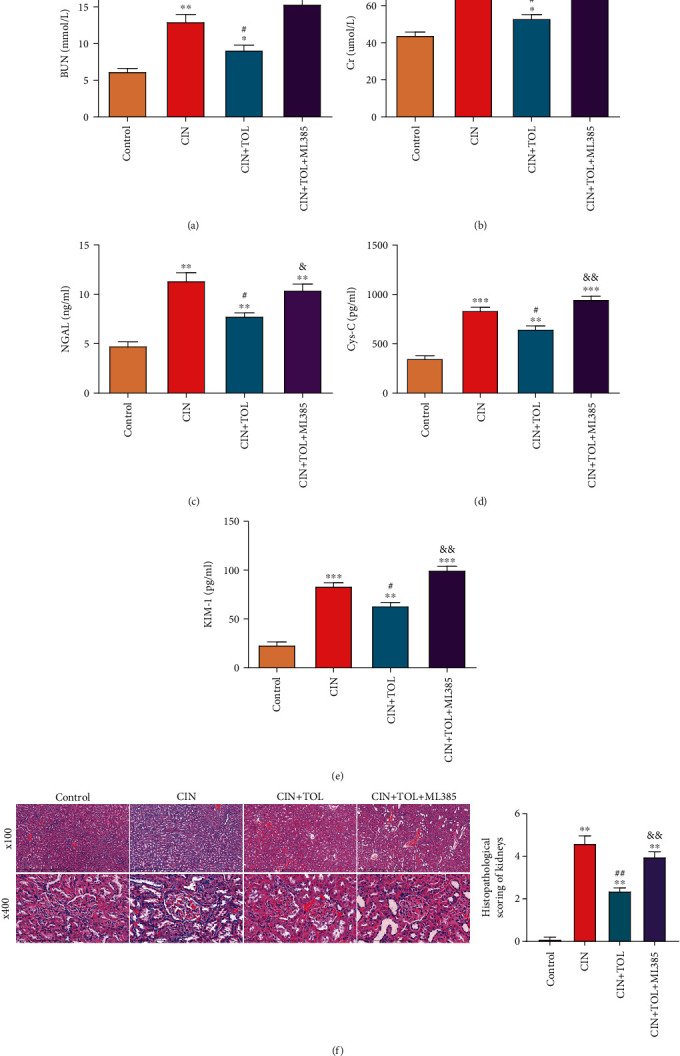
Effect of tolvaptan on contrast-induced acute kidney injury. Rats were divided into following four groups: The control group (control, *n* = 6), contrast-induced nephropathy group (CIN, *n* = 6), CIN rats administered tolvaptan group (CIN + TOL, *n* = 6), and CIN rats administered tolvaptan and Nrf2 inhibitor ML385 group (CIN + TOL + ML385, *n* = 6). Serum was collected from each group of rats. (a)–(c) Biochemical detection of levels of BUN (a), Cr (b), and NGAL (c). (d, e) ELISA detection of Cys-C (d) in rat serum and KIM-1 (e) levels in rat urine. (f) Representative H&E staining of kidney sections and histopathological scoring of renal injury. BUN: blood urea nitrogen; Cr: creatinine; NGAL: neutrophil gelatinase-associated lipocalin. Data are presented as means ± SD. ^∗^*P* < 0.05, ^∗∗^*P* < 0.01, and ^∗∗∗^*P* < 0.001 vs. control group; ^#^*P* < 0.05 and ^##^*P* < 0.01 vs. CIN group; &*P* < 0.05 and &&*P* < 0.01 vs. CIN + TOL group.

First, the effect of TOL on CI-AKI was evaluated. The results showed (Figures 2(a)–2(e)) that serum level of BUN, Cr, NGAL, and Cys-C and urinary level of KIM-1 were significantly increased in the CIN group compared with the control group (*P* < 0.05). In contrast, TOL pretreatment significantly decreased the level of BUN, Cr, NGAL, Cys-C, and KIM-1 (*P* < 0.05). In addition, Nrf2 inhibitor ML385 inhibited the effect of TOL.

H&E staining results showed severe kidney injury in the rats of CIN group, with tubular lumen congestion tubular vacuolization and unclear structure of brush border; additionally, CI-AKI rats showed necrosis and shedding of tubular cells and swelling and vacuolar degeneration of tubular epithelial cells. TOL pretreatment inhibited the injury above. However, ML385 attenuated the inhibitory effect of TOL on CI-AKI (Figure 2(f)). Collectively, TOL could attenuate CI-AKI, and the mechanism might be associated with the Nrf2 pathway.

### 3.2. TOL Inhibits Tubular Cell Apoptosis Caused by Contrast-Induced Kidney Injury

**Figure 3:**
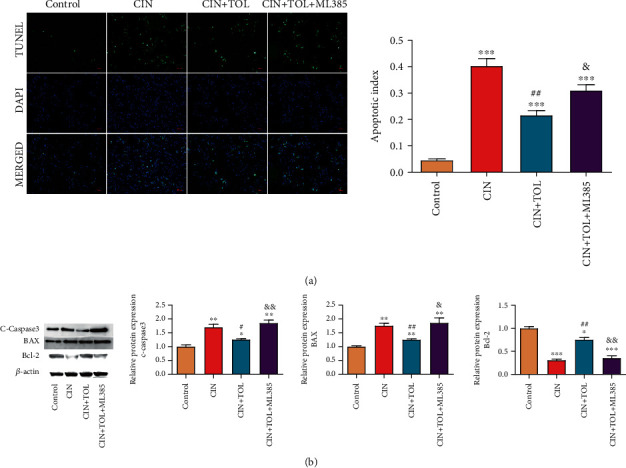
Effect of toraveptan on apoptosis in renal tubular cells caused by contrast-induced acute kidney injury. (a) TUNEL staining of rat kidney tissues to detect tubular cell apoptosis. (b) Western blot of rat kidney tissues to detect c-caspase 3, BAX, and Bcl-2 protein expression; CIN: contrast-induced nephropathy; TOL: tolvaptan; ML385: Nrf2 inhibitor. *N* = 6 per group. Data are presented as means ± SD. ^∗^*P* < 0.05, ^∗∗^*P* < 0.01, and ^∗∗∗^*P* < 0.001 vs. control group; ^#^*P* < 0.05 and ^##^*P* < 0.01 vs. CIN group; &*P* < 0.05 and &&*P* < 0.01 vs. CIN + TOL group.

It is reported that renal tubular apoptosis was a key mechanism of CI-AKI [23]. TUNEL staining and western blot were adopted to check the effect of TOL on renal tubular apoptosis induced by CI-AKI. In comparison with the control group, renal tubular epithelial cells in the kidney tissue of the CIN group showed TUNEL-positive signals, and the upregulation of c-caspase 3 and BAX and the downregulation of Bcl-2 were observed. After pretreatment with TOL in CI-AKI rats, the apoptotic signal of renal tubular cells was attenuated, c-caspase 3 and BAX expressions were significantly decreased, and the Bcl-2 expression was significantly increased, but ML385 inhibited the apoptosis-suppressing effect of TOL (Figures 3(a) and 3(b)*P* < 0.05). Taken together, TOL could inhibit CI-AKI-induced tubular cell apoptosis, and the Nrf2 signaling pathway might be involved in this process.

### 3.3. TOL Reduces CI-AKI Oxidative Stress and Mitochondrial Damage

**Figure 4:**
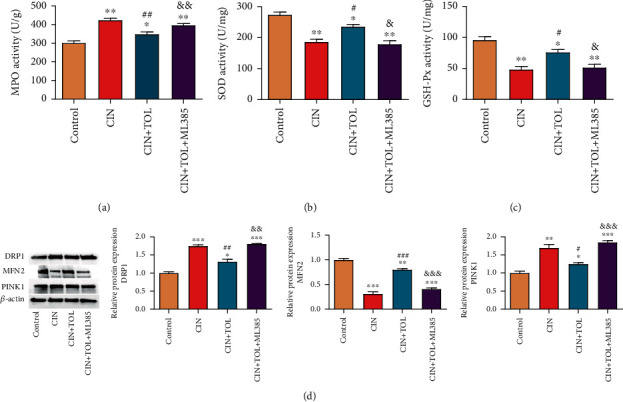
Effects of tolvaptan on contrast-induced acute kidney injury caused oxidative stress and mitochondrial function. Kidney tissues were collected from each group of rats.(a)–(c) The level of myeloperoxidase (MPO, (a)), superoxide dismutase (SOD, (b)), and glutathione peroxidase (GSH-Px, (c)) was measured. (d) The protein expression of DRP1, MFN2, and PINK1 in rat kidney tissues was detected by western blot. CIN: contrast-induced nephropathy; TOL: tolvaptan; ML385: Nrf2 inhibitor. *N* = 6 per group. Data are presented as means ± SD. ^∗^*P* < 0.05, ^∗∗^*P* < 0.01, and ^∗∗∗^*P* < 0.001 vs. control group; ^#^*P* < 0.05, ^##^*P* < 0.01, and ^###^*P* < 0.001 vs. CIN group; &*P* < 0.05, &&*P* < 0.01, and &&&*P* < 0.001 vs. CIN + TOL group.

Oxidative stress was also proved to be a key mechanism of CI-AKI [21]. Biochemical tests found that MPO level and the activities of SOD and GSH-Px in the kidney tissues of CI-AKI rats were significantly increased. TOL pretreatment significantly inhibited the level of MPO and upregulated the activities of SOD and GSH-Px in the kidney tissues of CI-AKI rats, but ML385 inhibited the effect of TOL (Figures 4(a)–4(c)*P* < 0.05). Further examination of protein expression of mitochondrial fission and fusion-related factors DRP1, MFN2, and PINK1 revealed that TOL downregulated DRP1 and PINK1 protein expression and upregulated MFN2 level; ML385 reversed the effects of TOL (Figure 4(d)). Collectively, TOL could attenuate oxidative stress in the kidney tissues of CI-AKI rats and protect mitochondrial function.

### 3.4. TOL Attenuates CI-AKI via Nrf2/Keap-1/HO-1 Pathway

**Figure 5:**
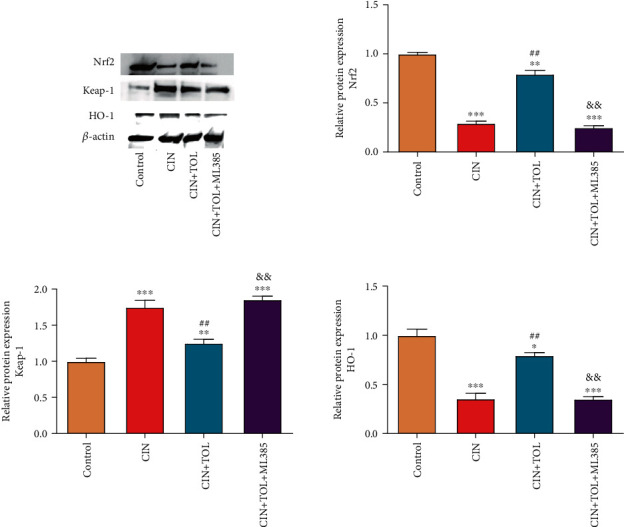
Effects of tolvaptan on the Nrf2/Keap-1/HO-1 pathway-related proteins. The kidney tissues of each group of rats were collected, the expression of Nrf2, Keap-1, and HO-1 proteins was detected by western blot, and the protein bands were analyzed in grayscale. Data are presented as means ± SD. ^∗^*P* < 0.05, ^∗∗^*P* < 0.01, and ^∗∗∗^*P* < 0.001 vs. control group; ^##^*P* < 0.01 vs. CIN group; &&*P* < 0.01 vs. CIN + TOL group.

The mechanism of TOL in inhibiting CI-AKI was further investigated by detecting the Nrf2 pathway-related proteins. The results showed that compared with the control group, the protein expression of Nrf2 and HO-1 was significantly downregulated, and the expression of Keap-1 was significantly upregulated in the CIN group. TOL could increase the Nrf2 and HO-1 expression and reduce the Keap-1 expression in the CIN rats. However, the addition of the Nrf2 inhibitor ML385 reversed the effects of TOL (Figure 5, *P* < 0.05). The results above suggested that TOL could attenuate CI-AKI by activating the Nrf2/Keap-1/HO-1 pathway.

## 4. Discussion

With the development of interventional radiologic technology in medicine, contrast media plays a pivotal role in the clinical practice [7]. However, relevant clinical studies have confirmed that the higher the dose of contrast media used in patients undergoing PCI, the higher incidence of CI-AKI and the more severe kidney injury [24, 25]. Therefore, it is of great significance to find drugs to prevent and treat CI-AKI. TOL, distinguished from conventional diuretics, is a highly selective vasopressin V2 receptor antagonist. TOL inhibits free water retention without adverse effects on renal hemodynamics [12, 26]. The description above suggests that TOL not only can promote diuretic effects but also can increase renal blood flow in patients with CI-AKI.

In this study, it is found that CI-AKI rats exhibited significant renal injury, such as significantly elevated serum BUN, sCr, NGAL, Cys-C and urinary KIM-1 level, and significant pathological changes in renal tubular tissue. However, TOL significantly reduced the level of the above molecules and improved the renal histopathological damage. Studies have shown that the level of BUN, sCr, and Cys-C was significantly increased after renal injury [27]. KIM and NGAL are also kidney-specific molecules [4]. In this study, the level of all above indicators was reduced after TOL pretreatment of CI-AKI, which suggested that TOL could significantly improve renal injury in CI-AKI.

The pathogenesis of CI-AKI is complex and has not yet been fully elucidated. And the main pathogenesis includes the following: contrast media enhances ROS formation and induced oxidative stress [28]; direct toxicity of contrast media to renal tubular epithelial cells aggravates apoptosis of renal tubular epithelial cells [29–31]; contrast media causes persistent ischemia and hypoxia in the renal medulla and then results in consequently damage to the kidney [32–34]. In our study, contrast media significantly increased oxidative stress and apoptosis of renal tubular cells in rat kidney tissues. However, TOL significantly attenuated renal tissue tubular cell apoptotic signals and inhibited the expression of apoptotic proteins cleaved-caspase 3 and BAX in CI-AKI rats. Meanwhile, TOL decreased MPO level and increased SOD and GSH-Px content in renal tissues, thereby inhibiting the occurrence of oxidative stress in renal tissues. The result of this study was in agreement with the findings of Bea et al. [18] Their study confirmed that paricalcitol attenuates renal injury by decreasing apoptosis and oxidative stress in renal tubular epithelial cells.

External inflammatory stimuli can lead to mitochondrial dysfunction, resulting in ATP consumption, increased ROS generation, calcium dysregulation, mitochondrial pore formation, release of proapoptotic proteins, and apoptosis [35]. DRPl can increase mitochondrial outer membrane permeability, decrease membrane potential, release cytochrome c, and then provoke apoptosis [36]. Downregulation or functional inhibition of MFN1 and/or MFN2 expression contributes to the reduction or even disappearance of mitochondrial tubular reticular structures, causing severe functional defects of cells; then, the cells show increased sensitivity to events that induce apoptosis, decreased mitochondrial membrane potential, and increased ROS generation and occur oxidative phosphorylation dysfunction and decreased ATP generation [37]. In this study, it is found that TOL could inhibit the expression of DRP1 and PINK1 while increase the MFN2 expression. The result of this study suggested that TOL played a renal protective role in CI-AKI rats by improving mitochondrial function.

The Nrf2 signaling pathway can be involved in the process of cellular antioxidative stress and can protect animal organisms from oxidative damage. And the Nrf2 signaling pathway is the most important oxidative stress pathway in the body, which has become a hot topic in the field of anti-oxidation in recent years [38]. Additionally, the Nrf2 signaling pathway can inhibit apoptosis and promote cell survival [39].

Some studies have reported that TOL can activate Nrf2 in renal cortical collecting duct cells and outer medulla of mouse kidneys and increase the expression of the antioxidant enzyme HO-1, thus activating the Nrf2/HO-1 antioxidant pathway [40]. Furthermore, in the LPS-induced AKI rat model, LPS injury increased the expression of Keap1 protein and inhibited the expression of Nrf2 and HO-1 [41]. The results in this study confirmed that TOL could upregulate Nrf2 and HO-1 expression and downregulate the Keap-1 expression in CI-AKI rats. It is also found that ML385, as a specific Nrf2 inhibitor [42], significantly inhibited the protective effect of TOL on the kidney. The results above confirmed that TOL could exert nephroprotective effects by activating the Nrf2/HO-1 antioxidant pathway. However, because CI-AKI was a complex pathophysiological process involving multiple factors, the subsequent roles of the Nrf2 pathway in the development of CI-AKI need to be investigated in more depth and in detail.

## 5. Conclusion

In summary, TOL can reduce oxidative stress response, improve mitochondrial function, activate the Nrf2/HO-1 antioxidant pathway, and reduce tubular cell apoptosis, thereby playing a renal protective role in CI-AKI. The results of this study can serve as a theoretical basis for the clinical use of TOL in the treatment of CI-AKI.

## Data Availability

The data used to support the findings of this study are available from the corresponding author upon request.
